# Advancing immune checkpoint blockade in colorectal cancer therapy with nanotechnology

**DOI:** 10.3389/fimmu.2022.1027124

**Published:** 2022-10-20

**Authors:** Zefan Liu, Yucheng Xiang, Yaxian Zheng, Xin Kang

**Affiliations:** ^1^ Department of General Surgery, First People's Hospital of Shuangliu District, Chengdu, China; ^2^ Department of Pharmacy, Third People’s Hospital of Chengdu, Chengdu, China

**Keywords:** colorectal cancer, immune checkpoint inhibitors, nanotechnology, drug delivery, tumor microenvironment

## Abstract

Immune checkpoint blockade (ICB) has gained unparalleled success in the treatment of colorectal cancer (CRC). However, undesired side effects, unsatisfactory response rates, tumor metastasis, and drug resistance still hinder the further application of ICB therapy against CRC. Advancing ICB with nanotechnology can be game-changing. With the development of immuno-oncology and nanomaterials, various nanoplatforms have been fabricated to enhance the efficacy of ICB in CRC treatment. Herein, this review systematically summarizes these recent nano-strategies according to their mechanisms. Despite their diverse and complex designs, these nanoplatforms have four main mechanisms in enhancing ICB: 1) targeting immune checkpoint inhibitors (ICIs) to tumor foci, 2) increasing tumor immunogenicity, 3) remodeling tumor microenvironment, and 4) pre-sensitizing immune systems. Importantly, advantages of nanotechnology in CRC, such as innovating the mode-of-actions of ICB, modulating intestinal microbiome, and integrating the whole process of antigen presentation, are highlighted in this review. In general, this review describes the latest applications of nanotechnology for CRC immunotherapy, and may shed light on the future design of ICB platforms.

## 1 Introduction

Colorectal cancer (CRC) is one of the leading causes of cancer-related deaths worldwide (1, 2). CRC induced 0.94 million deaths all over the world in 2020 (3). Moreover, more than 3.0 million new CRC cases are predicted in 2040 (2–4). To date, the standard treatment for CRC patients still remains surgical resection, but one-third of them are suffering from post-operative diseases. Challenges in the treatment of CRC are the formation of distant metastasis and the development of drug resistance (4). CRC gradually shows no response to traditional chemotherapeutics, thus novel therapies are urgently needed. Recently, therapy strategies that harness the host immune system against CRC seem to be beneficial for patients, especially those with high mutations (5, 6).

Tumor cells utilize immune checkpoint pathways to dampen T cell activation and evade attack by tumor-specific T cells (7). Immune checkpoint inhibitors (ICIs) competitively bind to checkpoint molecules and block the checkpoint-mediated suppression of the immune system (8). Monoclonal antibodies against checkpoint molecules such as programmed death 1 (PD-1)/programmed death ligand 1 (PD-L1) and cytotoxic T lymphocyte antigen 4 (CTLA-4) have yielded unprecedent success in CRC patients (9, 10). Some small-molecule compounds that directly inhibit PD-1/PD-L1 interaction (11, 12) and its regulatory proteins, such as bromodomain and extra-terminal domain (BET) (13, 14) and Src homology 2 domain containing protein tyrosine phosphatase (SHP2) (15, 16), as well as inhibitors of other immune checkpoints (CD47, CTLA-4, V-domain Ig suppressor of T-cell activation (VISTA)) (17, 18), are also under pre-clinical investigations. However, only highly mutated CRC patients (about 15% of total cases) that are mismatch repair deficient (dMMR) or exhibit high levels of microsatellite instability (MSI-H) can benefit from ICIs. In contrast, majority of CRC patients which are mismatch-repair-proficient (pMMR) or microsatellite instability-low (MSI-L) show negligible response to ICIs. Low tumor mutation and lack of immune cell infiltration are hypothesized as underlying mechanisms in these tumors (19–21). To date, nanotechnology provides powerful devices to detect, diagnose, and treat cancer (22, 23), and is considered as a potential strategy to reverse the immune resistance of CRC (24).

**Figure 1:**
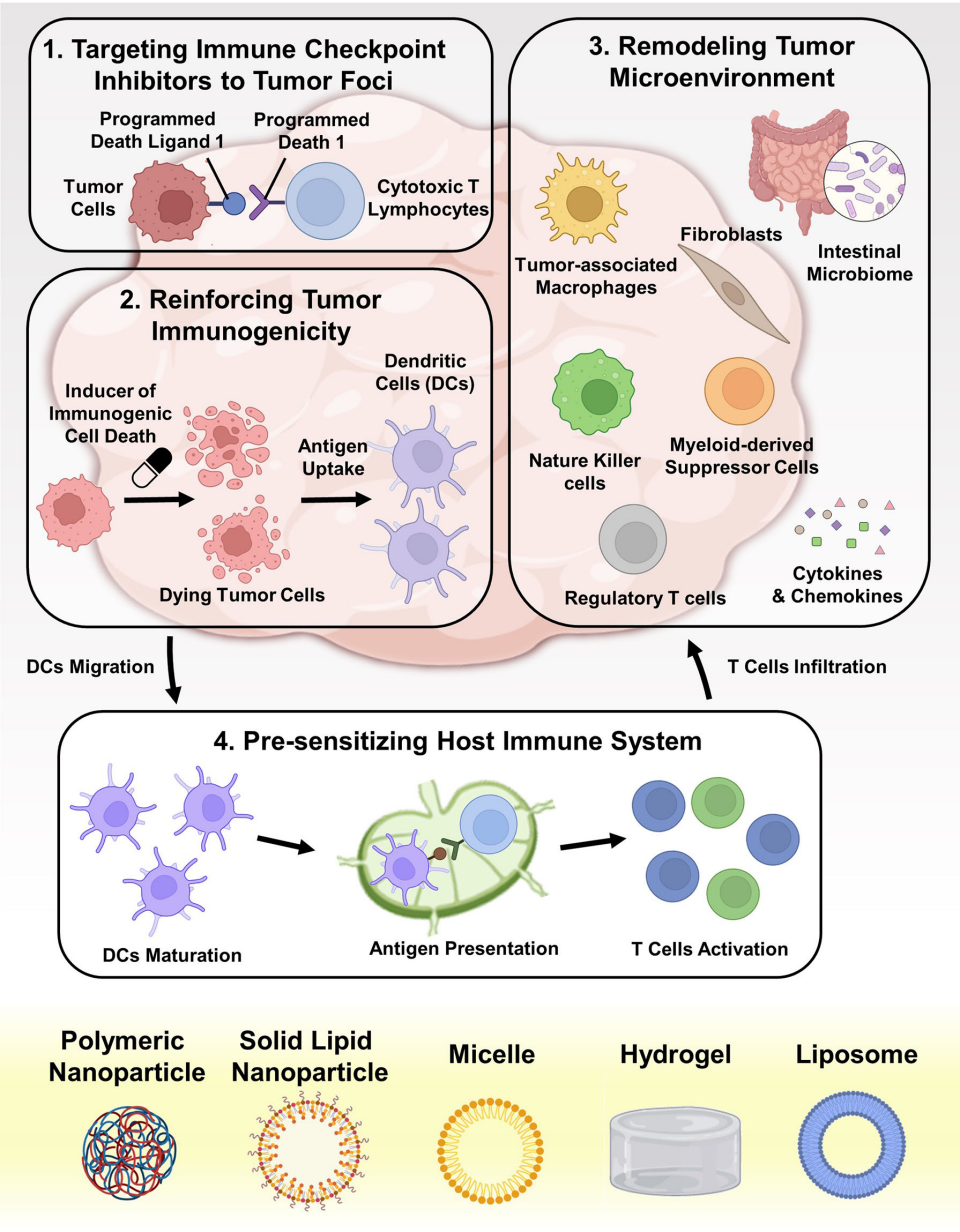
Schematic depiction of advancing ICB in CRC therapy with nanotechnology. The underlying mechanisms can be divided into four aspects: (i) targeted delivering various ICIs (such as antibodies, small molecules, peptides, and SiRNAs) into tumor foci; (ii) reinforcing the immunogenicity of dying tumor cells by cytotoxic agents and other drugs; (iii) remolding the immunosuppressive TME, including eliminating immunosuppressive factors and depleting immunosuppressive cells; (iv) pre-sensitizing the host immune system by delivering tumor vaccines and adjuvants to APCs.

Compared with conventional chemotherapeutics, nanomedicines not only exhibit superior tumoricidal ability and less side effects, but also present the potential to enhance immune checkpoint blockage (ICB) therapies (25) (1): By advancing the delivery of ICIs to CRC tumor sites, nanotechnology can directly enhance checkpoint blockade. During blood circulation, ICIs, especially small molecules, are difficult to accumulate in tumor beds. Macrophage-mediated phagocytosis system attenuates the delivery efficiency of ICIs (13). Even after entering CRC tissues, the amphiphilic cell membranes, lysosome degradation, and subcellular barriers (such as nuclear membranes, mitochondrial membranes, and endoplasmic reticulum membranes) also hinder the efficacy of ICIs (26). On the contrary, micro and nanosized particles can target to tumor tissue passively *via* the leaky tumor vasculature or actively *via* binding to receptors on tumor cell surface (27). Some systems can even deliver ICIs to certain subcellular compartments (28) (2). By regulating the cell death pathways, nanotechnology can transform immunologically tolerant cell corpse into immunogenic tumor vaccines, therefore amplifying ICIs efficacy (29, 30). Different from apoptosis, some engineered nano-systems can induce immunogenic cell death (ICD). In contrast to immune escape, tumor cells undergoing ICD will recruit antigen presenting cells (APCs), accelerate immune cells maturation, and initiate tumor antigen specific immune response *via* releasing various cytokines (31). Some other types of cell death such as ferroptosis, pyroptosis, and necroptosis might also be beneficial for increasing tumor immunogenicity (32–34) (3). By reprogramming immune-suppressive tumor microenvironment (TME), nanotechnology can revive the functions of ICIs (35–37). In order to respond to ICB, sufficient tumoricidal immune cell infiltration is necessary (38). In the progress of tumorigenesis, CRC tissues constantly release chemokines, cytokines, and exosomes as systemic factors to remold extracellular matrix (ECM) and recruit immunosuppressive cells, creating an immune desert milieu, which also terms as “cold” tumors (39, 40). Nanoparticles (NPs) can delivery agents that cut off the immune suppressive pathways, reversing malignant hallmarks in TME and reducing tumor-reside immunosuppressive cells (35, 41) (4). By facilitating the immune response against tumor-exclusive antigen pulses, NPs can potently provoke antigen specific immunity against CRC. As prophylactic or therapeutic interventions, NPs co-deliver antigens and immune-boosting adjuvants to host, pre-sensitizing immune system and systematically generating cytotoxic CD8^+^ T lymphocytes (CTLs) (42, 43). These four mechanisms are schematically illustrated in **Figure 1**.

In this review, we summarize the progress of nanotechnology applied to ICB-based CRC treatment in recent three years according to their underlying mechanisms. Particular advantages of nanotechnology in CRC immunotherapy, such as innovating the mode-of-actions of ICB, modulating intestinal microbiome, and integrating the whole process of antigen presentation, are highlighted. This review is expected to clarify the cross-interactions among drugs, materials, and organisms in CRC immunotherapy, and further improve the future design of ICB nanoplatforms.

## 2 Nanotechnology facilitates immune checkpoint blockade therapy

### 2.1 Targeting immune checkpoint inhibitors to tumor foci

Although ICIs have shown considerable clinical potency in prolonging survival of patients (44), growing evidences indicate that systemic administration of checkpoint blockade antibodies such as anti-PD-1 antibodies (αPD-1), anti-PD-L1 antibodies (αPD-L1), and anti-CTLA-4 antibodies (αCTLA-4) may cause undesirable autoimmune and inflammatory responses, such as colitis, dermatitis, and hypophysitis (45–47). Once happened, these unbearable adverse effects would seriously weaken therapeutic outcomes, or even fail the whole treatment (48, 49). Nanotechnology offers an attractive approach to bypass these side effects. Antibodies can be conjugated on or encapsulated in natural/artificial drug carriers, therefore avoiding antibody exposure in blood circulation (50, 51). In addition, nanosized drug delivery systems (DDSs) can passively/actively accumulate in solid tumors post systemic administration. Some DDSs can even be locally applied within tumor tissues (52, 53). Collectively, these formulations would remarkably elevate the selectivity of ICIs to tumors.

Checkpoint antibody-loaded NPs have been extensively studied. Early in 2010, Hellstrom et al. leverage functionalized mesoporous silica (FMS) to entrap αCTLA-4 (54–56). High drug loading and sustained drug release were achieved *via* adjusting the pore size of FMS, which minimize the risk of autoimmunologic toxicity (56). In addition to serving as drug reservoirs, antibody-conjugated NPs play significant roles in tumor theranostics. In colon tumor-bearing mice models, Popovtzer et al. demonstrated that the accumulation level of αPD-L1 conjugated gold NPs in tumors is an important parameter to predict the response of ICB therapy (57). Kang et al. attached methoxy poly (ethylene glycol) (MePEG) and chlorin e6 (Ce6) to Atezolizumab, a PD-L1 antibody, with a cathepsin B responsive linker (58). This immune checkpoint inhibitor nanocomposites (ICI NCs) avoided the ICI exposure in normal tissues, and exhibited tumor-activated fluorescence imaging (FI) and photodynamic therapy (PDT) on murine colon tumor #26 (CT26) tumor xenografts. Schneck et al. developed immuno-switch NPs that modified with αPD-L1 and anti 4-1BB antibodies on their surface (59). These dual-targeting NPs exhibited prolonged tumor retention than soluble free antibodies. After administration, immuno-switch NPs inhibited PD-L1 signal in tumor cells, and concurrently activated 4-1BB signal in CD8^+^ T cells, activating immune response against murine colon carcinoma 38 (MC38) in a two-pronged pathway.

**Figure 2:**
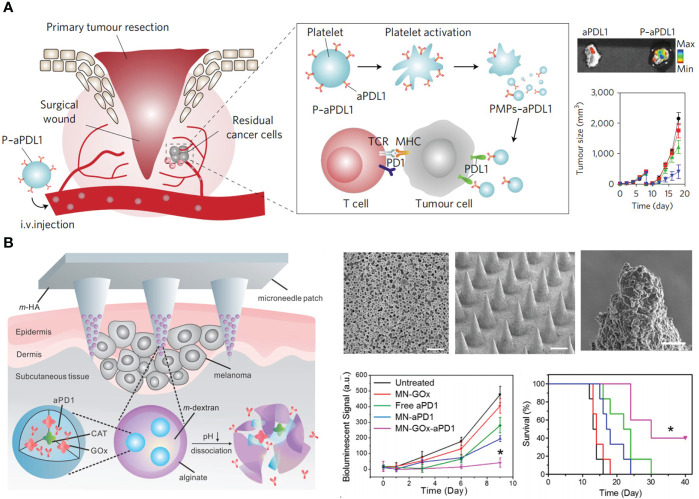
Nanotechnology targets ICIs to tumor foci to advance ICB in CRC therapy. **(A)** Left: schematic depiction of delivering αPD-L1 (aPDL1) to postsurgical tumor bed by platelets (P-aPDL1). Right top: P-aPDL1 sustainedly accumulated in tumor tissues. Right bottom: P-aPDL1 (blue curve) effectively inhibited tumor growth as compared with PBS-treated group (black curve). Reprinted with permission from reference (64). **(B)**. Left: schematic depiction of the composition of αPD-1 loaded microneedle (MN-GOx-aPD1). Right top: scanning electron microscope (SEM) images of αPD-1 loaded nanoparticles, microneedle patch, and magnified microneedle apex (from left to right). Right bottom: MN-GOx-aPD1 significantly inhibited tumor growth and prolonged mice survival. Reprinted with permission from reference (65). *p<0.05 versus untreated.

Solid tumors exhibited higher vascular density than normal tissues, and the wall of blood capillaries are highly leaky. Therefore, blood-circulating macromolecules (above 40 kDa) and NPs tend to extravasate and retain in tumor tissues (60). Moreover, the lymphatic drainage system is dysfunctional in tumors, which prevents the clearance of intra-tumoral NPs (61). This phenomenon is termed as the enhanced permeability and retention (EPR) effect. In general, the tumor accumulation efficiency depends on the blood circulation time of NPs and the tumor volume (62). NPs with prolonged blood circulation as well as decreased clearance by liver and kidney have more opportunity to be transported into tumor capillaries. And larger tumors have more disorganized vasculatures for NPs accumulation. Although this effect has been widely-acknowledged in mice models, its contribution to drug delivery in human is still controversial (62). The carrier with active tumor-homing capability is a better choice for ICI delivery. Wang et al. developed platelets as the carrier for αPD-L1 delivery (63, 64). Platelets have inflammation-targeting ability, and can secret various chemokines to boost T cell immunity, which is very favorable for delivering αPD-L1 into residual microtumors. Platelets binding with αPD-L1 (P-αPD-L1) aggregated in tumor tissue, turning into platelet-derived microparticles (PMPs) for tumor-specific antibody release (64). Treatment of P-αPD-L1 effectively prevented tumor metastasis and recurrence in incomplete tumor resection and thermal ablation (TA) models (**Figure 2A**).

Rather than systemic delivery, local application of antibodies within tumoral and peritumoral regions is an excellent approach to obviate the overactivation of the immune system (66). Melief et al. formulated αCTLA-4 into a water in oil emulsion composed of Montanide ISA-51 for subcutaneous (*s.c.*) injection in the tumor area (67, 68). This sustained-release platform had similar therapeutic consequences with systemic administration, but the dosage was only one-eighth of intravenous injection (*i.v.*), which contrastingly decreased antibody titers in serum and improved therapeutic safety. Similarly, Hubbell et al. prepared peptide-functionalized ICB antibodies for peritumoral injection (69, 70). A peptide derived from placenta growth factor-2 (PlGF-2_123–144_) showed super affinity with ECM. They showed the conjugation of PlGF-2_123–144_ elevated the tissue retention and decreased the plasma concentration of therapeutic antibodies (αPD-L1 and αCTLA-4), reducing the risk of systemic adverse effects, such as autoimmune diabetes. PlGF-2_123–144_ functionalized antibodies facilitated the infiltration of CD8^+^ and CD4^+^ T cells into tumors, resulting in delayed growth of primary and distance tumors. Gu et al. reported a microneedle (MN) patch for the transdermal delivery of αPD-1. Glucose oxidase (GOx) and αPD-1 were co-encapsulated into pH-sensitive dextran NPs, and these NPs were further loaded into hyaluronic acid MN arrays (65). GOx catalyzed the conversion of blood glucose to gluconic acid, forming a local acidic milieu for the self-disintegration of dextran NPs, leading to a sustained release of αPD-1 for three days. This simple and biocompatible platform could also be applied to co-deliver αPD-1 and αCTLA-4, achieving synergic antitumor effects (**Figure 2B**).

As the biological drugs, administration of antibodies may lead to infusion reactions and anti-drug antibodies (71, 72). Peptide-based ICIs, especially antagonists against PD-1 and PD-L1, are more preferable options. In comparison with antibodies, peptide drugs have deeper tumor penetration due to their low molecule weight (Mw). Given the lacking of Fc fragments, peptides exhibited lower immunogenicity and better safety. Moreover, peptide ICIs are more stable in structure, and cost less in manufacture, storage, and drug administration (73, 74). Even though, peptide ICIs have weaknesses including insufficient affinity, short body circulation, and a lack of tumor selectivity, which requires to be solved by suitable delivery systems. Kim et al. designed ferritin nanocages (PpNF) that displayed PD-L1- binding peptide (CLQKTPKQC) with multivalency on their surface (75). PpNF specifically accumulated in tumor tissues, and restored the antitumor activities of T cells. Notably, PpNF loaded with doxorubicin (DOX) had better tumor inhibition effect than αPD-L1 in CT26 tumor models (75). Huang et al. synthesized a liner polymer-drug conjugate of PD-L1 antagonistic peptide MSP (CPLGVRGSGQYASYHCWCWRDPGRSGGSK) (76). This polymer (P-MSP-DMA) intertwined with a mitochondria-targeted polymer-drug conjugate (P-D-R8MTS) *via* electrostatic interaction to form a nanocomplex (SNV). SNV specifically dissociated in tumors in response to the charge reversal of dimethylmaleic anhydride (DMA) group triggered by acidic TME. This nanoplatform integrated PD-L1 blockade with mitochondria-targeted induction of ICD, resulting in considerable inhibition of tumor growth and metastasis (76). NPs incorporated with PD-L1-binding peptides can also be combined with photothermal therapy (PTT), which was exemplified by Zhang et al. and You et al. (50, 52). Zhang et al. conjugated a PD-L1 antagonistic peptide (NYSKPTDRQYHF) on the surface of IR780-loaded NPs (aNP@IR780) (50). And You et al. co-encapsulated aN anti-PD-1 peptide ((SNTSESF)_2_ KFRVTQLAPKQIKE-NH_2_) and the hollow gold nanoshell (HAuNS) into NPs (AA@PN) (52). Both strategies simultaneously triggered tumor ablation and blocked PD-1/PD-L1 interaction between tumor cells and T cells, exhibiting an abscopal effect to suppress distant tumor growth in a bilateral CT26 tumor model.

Besides peptides, nuclei acid-based therapeutics against checkpoint molecules is another therapeutic alternative for ICB therapy. Wang et al. used poly (ethylene glycol)-block-poly (d,l-lactide) (PEG-PLA) and *N*-bis(2-hydroxyethly)-*N*-methyl-*N*-(2-cholesteryloxycarbonyl aminoethyl) ammonium bromide (BHEM-Chol) to encapsulate CTLA-4 siRNA (77). The prepared NPs (NP_siCTLA-4_) were capable to deliver siRNA cargos to both CD8^+^ and CD4^+^ T cells *in vivo*, facilitating their activation and proliferation. Ahn et al. prepared poly (lactic-co-glycolic acid) (PLGA) NPs to co-loading PD-1 siRNA and PD-L1 siRNA (siRNA@PLGA) (78). In the MC38 tumor model, they found the concurrent silencing of PD-1 and PD-L1 by siRNA@PLGA had better antitumor effect than single silencing of each one. Han et al. reported a nanoplatform with a novel PD-L1 binding aptamer, PL1 (51). In their design, PL1 single-stranded oligonucleotides were hybridized with folic acid (FA) and siRNA against proprotein convertase subtilisin-kexin type 9 (PCSK9) to obtain DNA tetrahedral nanoparticles (TDN-FA/PL1/Pcsk9-siRNA). TDN-FA/PL1/Pcsk9-siRNA were guided to CT26 CRC cells by FA, ensuring the synergy between PD-L1 blockade and Pcsk9 downregulation.

More than merely serving as a carrier for ICIs delivery and a platform for combinatory tumor immunotherapy, nanotechnology provides an opportunity to innovate the mode-of-action of ICB therapeutics. For example, Yang et al. leveraged lysosome-mediated receptor degradation to realize a durable PD-L1 downregulation. PD-L1 peptide antagonists (PPA, NYSKPTDRQYHF) were conjugated to the linear polymer composed of *N*-2 hydroxypropyl methacrylamide (HPMA) (79). By this way, PPA were transformed into a multivalent polymer-peptide antagonist against (MPPA). MPPA could gather and crosslink PD-L1 on tumor cell surface, biasing their trafficking to lysosome degradation and preventing their recycling to cell surface. This polymer-assisted receptor crosslinking strategy produced a long-lasting elimination of PD-L1 checkpoint, and strongly facilitated polymer-epirubicin (EPI) conjugate (KT-1) mediated chemo-immunotherapy. Nanotechnology may foster the druggability of ICI-like agents (79). Huang et al. designed a engineered PD-L1 trap as a novel ICB protein (53). In order to address the side toxicities of systemic PD-L1 blockade, the coding plasmid of PD-L1 trap was encapsulated in lipid-protamine-DNA nanoparticles (LPD). This system specifically distributed in tumor tissues, enabling the local production of the PD-L1 trap. In a CT26 murine colon tumor model, this strategy not only improved the tolerance of ICB therapy without inducing Th17 cells accumulation in spleen, but also achieved potent PD-L1 inhibition to potentiate oxaliplatin (OXA)-mediated chemotherapy (53).

### 2.2 Reinforcing tumor immunogenicity

In CRC, highly immunogenic tumors showed relatively good response to ICB-based immunotherapy (1, 80, 81). In contrast, the effect of ICIs in tumors with low immunogenicity requires further improvement (82). Recently, ICD has been reported to transform originally immunotolerant cell debris into immunogenic vaccines (83). ICD induced by anthracyclines was first reported by Guido Kroemer et al. in 2007 (84). Different from immune tolerant apoptosis, ICD can provoke the immune system to generate response against antigens from dead tumor cells, which is also known as “bystander effect” (85). Briefly, tumor cells undergoing ICD expose calreticulin (CRT) on the outer leaflet of the cell membrane, secreting adenosine triphosphate (ATP) and releasing high mobility group box 1 (HMGB1) into extracellular microenvironment (29, 30, 86). These markers facilitate APCs recruitment, antigen engulfment and presentation during immune initiation (87).

**Figure 3:**
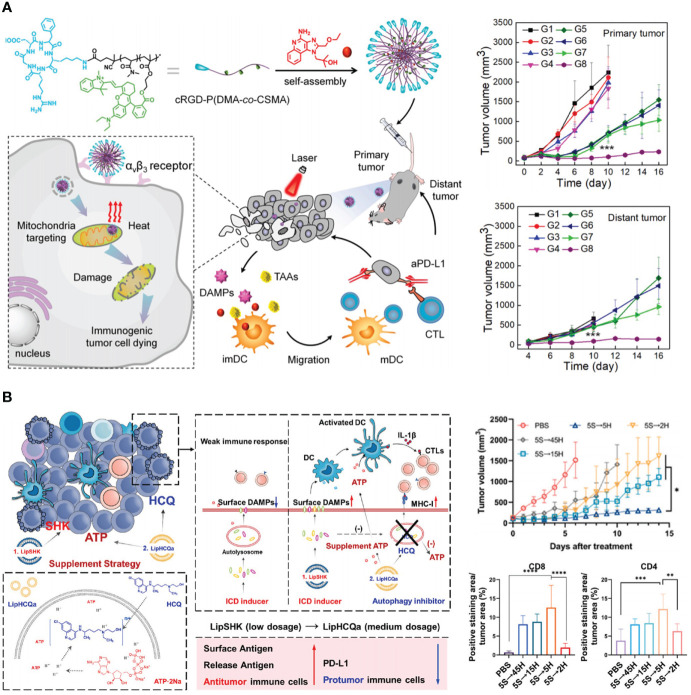
Nanotechnology reinforces tumor immunogenicity to advance ICB in CRC therapy. **(A)** Left: schematic depiction of mitochondria-targeted and photo-activated nanoparticles (R848@cRGD-PDCS) that triggered ICD to potentiate ICB therapy. Right: R848@cRGD-PDCS (G8) inhibited the growth of both primary tumor and distant tumor in combination with αPD-L1. Reprinted with permission from reference (89). ***p<0.001 versus G1. **(B)** Left: schematic depiction of the mechanism of recovering tumor immunogenicity by autophagy inhibition. Right: impacts of different autophagy inhibition therapies on tumor growth and the percentage of CD8^+^ and CD4^+^ T cells. Reprinted with permission from reference (90). *p<0.05, **p<0.01, ***p<0.001, ****p<0.0001.

DOX is often administrated along with other chemotherapeutics to elevate efficacy. Although DOX is found to facilitate effector T cells infiltration and synergize with ICIs *via* ICD induction, applications of free DOX are still hindered by cardiac toxicity and unsatisfactory tumor accumulation (29). Jeffrey A. Hubbell et al. reported a collagen-binding serum albumin platform for advanced colon carcinoma therapy (88). Serum albumin (SA) based carrier can passively deliver drug to tumor sites *via* the extravasation through pathological vasculature. To further endow SA active targeting capacity, a collagen-binding domain (CBD) was fused recombinantly to give CBD-SA. Lastly, DOX was loaded to CBD-SA *via* a pH-sensitive linker (DOX-CBD-SA). Surprisingly, when combing with αPD-1, a complete eradication of MC38 colon carcinoma was observed. To understand the underlying mechanism, T cells and natural killer (NK) cells in treated tumors were extracted. The numbers of CD8^+^ T cells, CD4^+^ T cells and NK cells per unit tumor mass increased after DOX-CBD-SA treatment. The increased tumor-infiltrating lymphocytes subsequently potentiated therapeutic efficiency of immune-checkpoint blockade. Generally, DOX-CBD-SA can potently kill tumor cells and simultaneously stimulate host antitumor immunity, decreasing adverse events. To further improve the immunogenicity, subcellular level targeting strategy was considered. Mitochondria is one of the most important organelles and serves as the source of damage associated molecular patterns (DMAPs) such as ATP, heat shock protein 70 (HSP70), and HSP90. The released DMAPs facilitate the presentation of tumor-associated antigens. Zhan et al. engineered a mitochondria-targeted polymeric nanoparticle (R848@cRGD-PDCS) (89). Under near-infrared irradiation exposure, mitochondria were destroyed by photothermal-mediated hyperthermia, causing the release of tumor-associated antigens and DMAPs. αPD-L1 therapy showed limited inhibitory effects in tumor growth, but the combination with R848@cRGD-PDCS (under irradiation) exhibited favorable ability to eradicate primary tumors and prevent metastasis (**Figure 3A**).

Chemotherapeutics (such as OXA and DOX) and PDT were reported to induce ICD synergistically. Lin et al. proposed a core-shell nanoscale coordination polymer (NCP@pyrolipid) which not only directly eliminated tumor cells but also promoted the checkpoint blockade immunotherapy (91). In the study, OXA in the core and the photosensitizer pyropheophorbide-lipid conjugate (pyrolipid) in the shell synergistically eradicate cancer cells, resulting in robust ICD and subsequent abscopal effects. Moreover, after integrating PD-L1 blockade with NCP@pyrolipid, tumor regression was observed in both light-irradiated primary tumors and distant tumors without light-irradiation, indicating that a potent tumor-specific immunity was evoked. The authors observed increased portions of antigen-specific CTLs in the CRC-bearing mice injected with NCP@pyrolipid (with irradiation) plus αPD-L1. The immunogenic environment induced by both OXA and PDT remarkably enhanced PD-L1 therapy *via* spurring systemic antitumor immune response. Yu et al. reported an prodrug-based polymeric nanoparticle to realize optimal administration of OXA combining with PTT (92). Besides, fluorescence -guided PTT can further enhance tumor immunogenicity and release drug in a spatiotemporally controllable way. A donor-spacer-acceptor-space-donor (D-S-A-S-D) type fluorophore was farther inserted to improve immunogenicity and amplify the efficacy of αPD-L1 therapy. This combinatory chemo/photothermal therapy with PD-L1 blockade (PBOXA@TQTCD+L-αPDL1) was tested *in vivo*. Results revealed that the combinatory therapy not only inhibit tumor growth much more potently than αPD-L1 therapy alone, but also improve the survival rate of mice with tumor. When it comes to the tumor recurrence inhibition, the central memory CD8^+^ T cells (T_CM_) in spleen representing long-term immune memory was analyzed. T_CM_ ratio in the combinatory therapy was at least two times higher than that of αPD-L1 alone, indicating the activation of a long-term immune surveillance against tumor recurrence. This combinatorial therapy might enlighten clinical CRC management.

However, the PDT or PTT is a localized therapy and restricted by light penetration. Alternatively, Lin et al. reported another tactic that using reactive oxygen species (ROS) based chemotherapeutic to induce potent ICD and synergize with OXA. The author engineered self-assembled coordination polymer nanoparticles (OxPt/DHA) loading OXA in the core and ROS-generating dihydroartemisinin (DHA) in the shell for CRC treatment (93). In a tumor rechallenge experiment, mice vaccinated with OxPt/DHA-treated cells showed a potent immune resistance against live MC38 cells and no tumor formation was observed. The efficacy of OxPt/DHA combining with α-PD-L1 blockade therapy was tested in tumor models of CT26 and MC38 on immunocompetent BALB/c and C57BL/6 mice, separately. In both CT26 and MC38 models, the α-PD-L1 therapy alone failed to control tumor growth. In the contrary, all of the tumors treated with OxPt/DHA plus α-PD-L1 regressed and ultimately disappeared on days 40~50. Until 120 days, no recurrence was found. Results revealed that OxPt/DHA is a potential clinical candidate to synergize with ICIs.

Besides inducing ICD *in situ* in tumor sites to enhance immunogenicity and amplify immune checkpoint blockade (ICB) therapy, immunogenically dying tumors cells themselves can also be transformed into a powerful platform for cancer vaccination. Moon et al. have manufactured dying tumor cells surface-modified with adjuvant-contained NPs (94). Results revealed that dying tumor cells undergoing ICD could be further filled with adjuvant nano-depots to successfully initiate antigen cross-presentation by dendritic cells and activate potent antigen-specific CD8α^+^ T cells in mice model bearing CRC. Additionally, the combinatory regimen using this whole tumor-cell vaccination and immune checkpoint inhibition resulted in a complete tumor eradication in about 78% of mice inoculated with CT26. A long-term immunity was also observed, indicating the potential to prevent tumor recurrence. This strategy might shed light on “personalized” therapy which is tailored according host’s own tumor cells. The inflammatory microenvironment after surgery and residual tumor “seeds” were responsible for post-operative metastasis. To solve this dilemma, Li et al. embedded autologous cancer cells succumbing to ICD and anti-inflammatory drug dexamethasone in hydrogel, the hydrogel could be injected into a resection site, in which it was rapidly solidified and gradually degraded (95). The dying cells provided a whole array of tumor-associated antigens and became highly immunogenic vaccines which enabled antigen specific immunization. After combining with αPD-L1 therapy, a complete tumor regression was observed, which might be attributed to their complementary functions to evocate and unleash tumoricidal T cells. This strategy provides a novel option for inhibiting metastasis after surgery.

Autophagy refers to the process by which cells degrade their constituents by autophagosomes. Autophagy is necessary in sustaining and modulating cell homeostasis. In addition, autophagy facilitates the release of ATP from lysosome in ICD inducing, promoting antitumor immune response. However, autophagy can destroy tumor-associated antigens, therefore attenuating antitumor immunity. To overcome this difficulty, Wang et al. designed a liposome named as LipHCQa which encapsulated shikonin (ICD inducer), hydroxychloroquine (autophagy inhibitor), and ATP for the treatment of colon cancer (90). This compensatory liposome showed enhanced immune infiltration when compared with shikonin loaded liposome alone, indicating the importance of blocking autophagy on ICB amplification (**Figure 3B**).

Specific series of intracellular suicide process was named as programmed cell death (96, 97). In the past few decades, apoptosis had been assumed as the sole modality of programmed cell death (84, 98). Recently, several other pathways of programmed cell death were identified, such as ferroptosis and pyroptosis (99, 100). These particular cell death pathways might be used to enhance the immunogenicity of tumor cells and synergize with ICB (101). Since firstly proposed in 2012 by Stockwell and co-workers, ferroptosis has attracted numerous attentions in the field of oncology and biochemistry (102, 103). Ferroptosis is an iron and ROS dependent dell death. Cells undergoing ferroptosis showed increased lipid peroxidation products and ROS that is derived from iron metabolism. Han et al. designed core-shell nanoparticles (ZnP@DHA/Pyro-Fe) loaded with a cholesterol derivative of dihydroartemisinin and pyropheophorbide-iron (Pyro-Fe) to potentiate CRC immunotherapy *via* inducing ferroptosis. ZnP@DHA/Pyro-Fe treated cancer cells showed increased DAMPs release and result into intra-tumoral immune cell infiltration (104). Further combination with αPD-L1 checkpoint blockade led to better therapeutic effect. Different from caspase-dependent apoptosis, necroptosis is featured by expanded cell volume, organelle swelling, cell membrane fracture, and leaking of intracellular components. Nowadays, mixed-lineage kinase domain like protein (MLKL), receptor interacting protein-1 (RIPK1), and RIPK3 pathways were thought to be essential in tumor necrosis factor-α (TNF-α) mediated necroptosis. Compared with poorly immunogenic apoptosis failing to activating antitumor immunity, necrosis showed great potential in priming immune response due to the increase immunogenicity (105). Sun et al. prepared dimethyl fumarate loaded star-PCL-azo-mPEG (sPCEG-azo) polymeric micelles (106). The micelles are colon-targeted and induce necroptosis in colon cancer cells *via* a mechanism characterized with increased ROS. The elevated ROS generation result in immunogenicity and contribute to antitumor immunity, which may further augment ICB. Another necroptosis-inducible nanoparticle was reported by Park et al. The nanobubbles (NBs) contains Ce6 as the sonosensitizer and perfluoropentane as the gas precursor (107). After ultrasound exposure, NBs could disintegrate plasma membrane and lead to damage-associated molecular patterns release, inducing acoustic cavitation mediated necroptosis and ROS-mediated tumor regression. The NBs promoted antitumor immunity *via* accelerating dendritic cells maturation and CD8^+^ T cells activation. Further combinatory regimen including PD-L1 blockade plus NBs even led to complete eradication of primary CT26 tumor and metastasis.

### 2.3 Remodeling tumor microenvironment

TME is an intricate milieu including tumor and immune cells, bacteria, as well as multiple soluble signal mediators (108, 109). All of them contribute to the distinct physiological characteristics (hypoxia, acidity, inflammation, and immune escape) of TME. Mounting evidence reveal that the heterogeneity of TME is an important factor for the low responsiveness of ICB therapies, and reversing immunosuppressive TME is very promising to overcome ICI resistance (110). Considering the close interaction between components in TME, nanoplatforms that counteract these suppressors in multi-pronged ways are very promising (36, 111, 112). Nanoplatforms that potentiated ICB against CRC by modulating TME are summarized in **Table 1**.

**Table 1:** Summary of nanoplatforms that remolds TME to advance ICB in CRC therapy.

Target	Nanoplatform	Route of administration	Mechanism of TME remolding	Reference
Hypoxia	perfluorocarbon-loaded liposome (PFC@lipo), hemoglobin-loaded liposome (Hb@lipo), hypoxia inducible factor-1α (HIF-1α) inhibitor (PX-478)	Liposomes: *i.v.* Free drug: *i.p.*	PFC@lipo and Hb@lipo loaded and delivered oxygen to tumor, PX-478 inhibit the hypoxia signal pathway	(113)
cyclooxygenase-2 (COX-2)	Self-assembled polymeric prodrug of aspirin (P3C-Asp)	*i.v.*	P3C-Asp released aspirin in response to high ROS level and specifically inhibited COX-2 in TME	(114)
Intestinal microbiome	lipid–protamine–DNA nanoparticles (LPD) that loaded the plasmid encoding lipopolysaccharide (LPS)-binding protein	*i.v.*	The nanoparticles accumulated in tumor, and then expressed protein which depleted LPS in CRC tissues	(115)
	M13 bacteriophage coated with silver nanoparticles (M13@Ag)	*p.o.*	The M13 specifically bound with *Fusobacterium nucleatum* (*Fn*) and then Ag nanoparticles eliminated *Fn*	(116)
Indoleamine 2,3-dioxygenase 1 (IDO-1)	Boolean logic prodrug nanoparticles (BLPNs) incorporated with IDO-1 inhibitor NLG919 and photosensitizer pheophorbide a (PPa)	*i.v.*	BLPNs released NLG919 in response to the high glutathione (GSH) level in tumor cells, which inhibited the metabolism of tryptophan	(117)
	Cationic lipid-assisted nanoparticles loaded with siRNA of IDO-1 (CLAN*_siIDO1_*) and oxaliplatin (OXA)	*i.v.*	CLAN*_siIDO1_* accumulated in both tumor tissues and tumor-draining lymph nodes (TDLNs), downregulating IDO-1 that upregulated after OXA treatment in these two tissues	(118)
phosphatidylserine (PS) externalization	Annexin A5-modified and neoantigen-loaded nanoparticles (AnnV_PLGA(Nbea)_NPs) and cisplatin	AnnV_PLGA(Nbea)_NPs: *i.v.* Cisplatin: *i.p.*	The surface Annexin A5 of AnnV_PLGA(Nbea)_NPs blocked the immunosuppressive effects of PS on dying tumor cells treated by cisplatin	(119)
Tumor-associated macrophages (TAMs)	Pexidartinib-loaded nanoparticles (PLX-NPs) and αPD-L1 conjugated platelets (P-aPD-1)	*s.c.* after incorporation together into hydrogel or *i.v.* separately	Pexidartinib blocked the colony-stimulating factor 1 receptors (CSF1R) on TAM surface and depleted TAM	(120)
	Red blood cell (RBC) membrane-coated *Porphyromonas gingivalis* (*cmPg*)	Intra-tumoral injection	*Pg* promoted the polarization of TAM towards anti-tumoral M1 phenotype	(121)
Signal pathways and bacteria	polymeric metformin (Polymet)	*p.o.*	Polymet activated adenosine 5’-monophosphate activated protein kinase (AMPK) pathway, inhibited mammalian target of rapamycin (mTOR) pathway, and increased anti-tumoral *Lactobacillus* in CRC tissues	(122)
Hypoxia, IDO-1, and myeloid-derived suppressor cells (MDSCs)	MnO_2_ mineralized nanocage co-encapsulated with IDO-1 SiRNA and gemcitabine (GEM), the surface of nanocage was modified with antibody against PD-L1/CD47	*i.v.*	MnO_2_ catalyzed the generation of oxygen in TME, inhibited HIF-1α and promoted M1 macrophage polarization; SiRNA silenced IDO-1 and suppressed regulatory T cells (Tregs); GEM eliminated MDSC	(123)

i.v., intravenous injection; i.p., intraperitoneal injection; p.o., oral administration; s.c., subcutaneous injection.

#### 2.3.1 Hypoxia

Hypoxia reduced the therapeutic efficiency of ICIs in many aspects. It increased the expression of PD-L1 and CTLA-4 *via* hypoxia inducible factor-1α (HIF-1α) pathway, and weakened antigen presentation of APCs (124, 125). You et al. adopted three strategies to alleviate hypoxia (113): (i) directly deliver oxygen into tumors by a perfluorocarbon-loaded liposome (PFC@lipo); (ii) directly deliver oxygen into tumors by a hemoglobin-loaded liposome (Hb@lipo); and (iii) indirectly inhibit HIF-1α in tumors by a small molecular inhibitor PX-478. They found systematic administration of Hb@lipo was the most ideal strategy to combine with αPD-1 for the treatment of murine triple-negative breast cancer (4T1) and CT26 tumors. Due to the hypoxia milieu, tumor cells are glycolytic and produce plenty of lactate as the metabolite, resulting in anergy of tumor-infiltrated immune cells. Dhar et al. developed a mitochondria-targeted NPs (T-Mito-DCA-NPs) for the delivery of dichloroacetate (DCA), an inhibitor of pyruvate dehydrogenase kinase 1 (PDK1) (126). This formulation selectively inhibited tumoral glycolysis without affecting immune cells. As a result, T-Mito-DCA-NPs significantly elevated the intra-tumoral infiltration of CD8^+^ and CD4^+^ T cells and downregulated the expression of checkpoint molecules including PD-1, CTLA-4, LAG3, and Tim3 on their surface. Combination of mitochondria-targeted DCA and αPD-1 effectively improved the infiltration of CD8^+^ and CD4^+^ T cells in CT26 tumors.

#### 2.3.2 Inflammation

Inflammation is an important hallmark in TME that promote tumorigenesis, expansion, metastasis, and immune escape (127). Tumor cells highly expressed cyclooxygenase-2 (COX-2) and secreted a large amount of prostaglandin E2 (PGE2) to recruit myeloid-derived suppressor cells (MDSCs) (128, 129). MDSCs generate several immunosuppressive cytokines, such as ROS, transforming growth factor-β (TGF-β), and interleukin 10 (IL-10), leading to failure of ICB therapy (130–132). Chen et al. synthesized a self-assembled polymeric prodrug (P3C-Asp) of aspirin, a classical non-steroidal anti-inflammatory drug (NSAID) (114). P3C-Asp released aspirin in response to high ROS level in tumor tissues, decreasing PGE2 secretion and reversing tumor immunosuppression. Combination therapy of P3C-Asp+αPD-L1 eradicated CT26 tumors in 100% of mice.

#### 2.3.3 Intestinal microbiome

**Figure 4:**
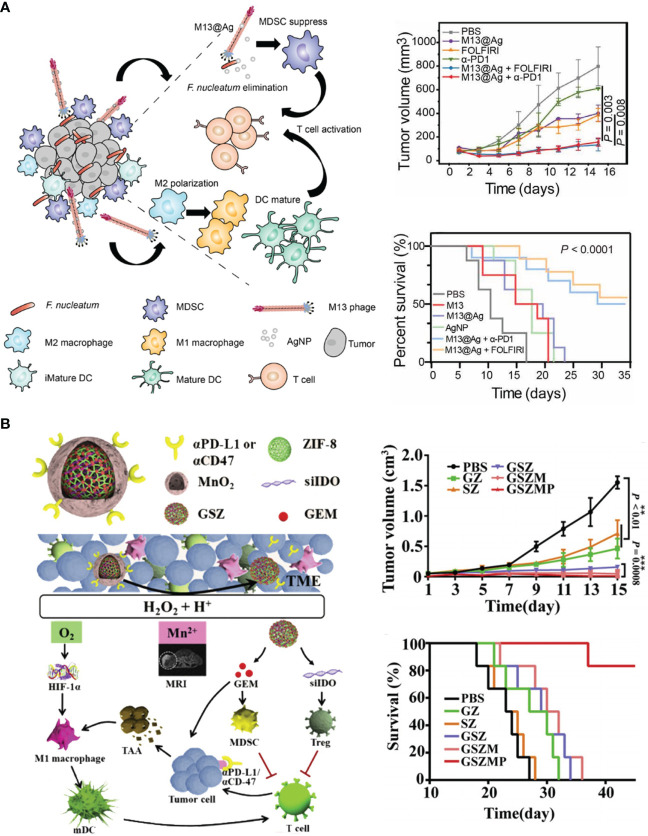
Nanotechnology remolds TME to advance ICB in CRC therapy. **(A)** Left: schematic depiction of engineered bacteriophage (M13@Ag) that regulated intestinal microbiome to modulate TME against CRC. Right: M13@Ag significantly inhibited tumor growth and prolonged mice survival in combination with αPD-1. Reprinted with permission from reference (116). **(B)** Left: schematic depiction of the composition of versatile nano-modulator (GSZMP) and its mechanism in potentiating ICB therapy. Right: GSZMP potently inhibited tumor growth and prolonged mice survival. Reprinted with permission from reference (123).

Different from other cancers, the progress of CRC is closely associated with intestinal microbiome. The species and abundance of intestinal microbiome highly affected the balance in GI track, contributing to several gut diseases, such as colitis, fibrosis and CRC (133). *Fusobacterium nucleatum*, *Bacteroides fragilis*, and *Escherichia coli* are the main pathogenic bacteria in gut that promote tumor progression and hinder the responsiveness of CRC to αPD-L1 therapy (134–136). Bacteria colonized in cancerous GI tracts produced massive endotoxin, also known as lipopolysaccharide (LPS). LPS accelerated the growth and liver metastasis of colorectal tumors *via* Toll-like receptor 4 (TLR4) and nuclear factor kappa-B (NF-κB) pathway (137–139). Moreover, chemotherapeutic agents are able to disrupt the mucus barrier in gastrointestinal (GI) track, facilitating the colonization and invasion of gut bacteria (140). Huang et al. designed an LPS-binding fusion protein as the trap to deplete LPS in orthotopic CRC tissues (115). For tumor selectivity, they encapsulated the coding sequence of LPS trap protein into lipid–protamine–DNA nanoparticles (LPD). This system passively accumulated in CT26 tumors, enabling LPS trap protein expressed within malignant tissues. They found LPS trap treatment elevated the infiltration of T cells, which favored ICB therapy. Combinatory treatment of the LPS trap and αPD-L1 not only retarded the growth of orthotopic CT26-FL3 tumors but also inhibited their spontaneous liver metastasis. Zhang et al. identified *Fusobacterium nucleatum* (*Fn*) as pro-tumoral gut bacteria that restricted T cell infiltration and enriched MDSCs in CRC tissues (116). They screened a *Fn*-binding M13 bacteriophage by phage display technology and modified antibacterial silver nanoparticles (AgNP) on its surface. The obtained M13@Ag could specifically eliminate *Fn* in GI track to reduce MDSCs in TME, and facilitate antigen presentation due to its intrinsic immunogenicity. In orthotopic CRC models, combination therapy of M13@Ag and αPD-1 significantly improved the overall survival of mice (**Figure 4A**).

#### 2.3.4 Ido-1

Indoleamine 2,3-dioxygenase 1 (IDO-1) is highly overexpressed on tumor cells (141, 142). It is a rate-limiting enzyme in the kynurenine pathway that convert tryptophan to kynurenine (143). The accumulation of kynurenine in TME contributed to dendritic cells (DCs) deactivation, CTL apoptosis, and regulatory T cells (Tregs) increment (144, 145). Due to its key role in tumor immunosuppression, IDO-1 is termed as the “metabolic immune checkpoint” (118). Inhibiting IDO-1 have been demonstrated to potentiate ICD-based chemo/photo-immunotherapy and PD-1/PD-L1 blockade therapy, but the therapeutic consequence is strongly relied on well-designed delivery strategies. Yu et al. developed boolean logic prodrug nanoparticles (BLPNs) that logically gated by matrix metalloproteinase (MMP), acid, and glutathione (GSH) to release photosensitizer pheophorbide a (PPa) and IDO-1 inhibitor NLG919 to treat CT26 tumors (117). PPa induced ICD to trigger T cell response, which was further amplified by NLG919. Wang et al. accomplished the concurrent inhibition of IDO-1 in both tumors and tumor-draining lymph nodes (TDLNs) by siRNA-loaded cationic lipid-assisted nanoparticles (CLAN*_siIDO1_*) (118). They found OXA treatment aggravated IDO-1 overexpression in tumors and TDLNs, which was in accordance with other immune checkpoints like PD-L1 and CTLA-4. CLAN*_siIDO1_* accumulated in TDLNs and tumors, downregulated IDO-1 in both tissues, and improved tumor inhibition by OXA in the CT26 colon cancer model.

#### 2.3.5 PS externalization

Chemotherapy triggers phosphatidylserine (PS) externalization on the surface of tumor cells undergoing apoptosis (146, 147). In line with canonical immune checkpoints, the exposed PS consolidated the immunosuppressive TME, restricted the phagocytosis of APCs and upregulated the expression of PD-L1 (148, 149). Recently, Park et al. developed annexin A5-labeled NPs (AnnV_PLGA_NPs) as the inhibitor to this innate immune checkpoint. Mutant neoantigen peptides (Nbea, PAPRAVLTGHDHEIVCVSVCAELGLVI) were loaded in NPs (AnnV_PLGA(Nbea)_NPs) to elicit antigen-specific antitumor immunity (119). In company with cisplatin (Cis)-mediated chemotherapy, AnnV_PLGA(Nbea)_NPs spurred the infiltration of immune-activate cells and the secretion of pro-inflammatory cytokines, while depleting immune-suppressive MDSCs and Tregs and decreased the production of anti-inflammatory cytokines at the same time. The immunostimulatory effect of AnnV_PLGA(Nbea)_NPs can be amplified by αPD-L1, and the triple-therapy of Cis + AnnV_PLGA(Nbea)_NPs + αPD-L1 resulted in noticeable rejection of CT26 tumor growth.

#### 2.3.6 TAMs

Tumor-associated macrophages (TAMs) represented a large population in intra-tumoral immunosuppressive cells (150). Generally, TAM can be divided into anti-tumoral M1 phenotype and pro-tumoral M2 phenotype *via* their different markers. M1 macrophages not only killed tumors in an innate manner, but also presented tumor antigen to T cells and activated adaptive tumor immunity (151). M2 macrophages are the major TAMs in immunosuppressive tumors, such as triple-negative breast cancer (TNBC) and CRC (152). They affected the function of tumor and immune cells in TME by secreting vascular endothelial growth factor (VEGF), IL-10, TGF-β, and arginase-1 (153). There are three paradigms for targeting TAMs: (i) inhibiting TAMs recruitment, (ii) depleting pre-existing TAMs, and (iii) re-educating TAMs from pro-tumoral M2 macrophages to anti-tumoral M1 phenotype (151, 152). Results of Hu et al. revealed that locally depleting TAMs in postsurgical tumor beds by Pexidartinib-loaded nanoparticles (PLX-NPs) created an appreciable condition for the local and systemic PD-1 blockade therapy (120). Pexidartinib eliminated TAMs by blocking colony-stimulating factor 1 receptors (CSF1R) on TAM surface. Alginate hydrogel incorporated with PLX-NP (PLX-NP@Gel) decreased F4/80^+^ macrophages and increased IFN^+^CD8^+^ CTLS in tumor beds. αPD-1 conjugated platelets (P-aPD-1) were co-encapsulated into hydrogel for local implantation (PLX-NP-P-aPD-1@Gel) or systematically injected (PLX-NP- @Gel+P-aPD-1). Both regimes considerably inhibited post-surgery tumor recurrence in murine melanoma (B16F10), CT26, and 4T1 tumor models. Conventional TAM-modulating strategies employed small molecules, peptide, antibodies, and nuclei acids, while Zhou et al. reported a bacteria-based approach to repolarize TAMs (121). They prepared a red blood cell (RBC) membrane-coated formulation of *Porphyromonas gingivalis* (*cmPg*). *Porphyromonas gingivalis* (*Pg*) not only promoted the conversion of TAMs to M1 phenotype, but also secreted melanin for tumor PTT. With the help of PD-L1, *cmPg* retarded the growth of primary and secondary CT26 colon tumors.

#### 2.3.7 Multiple targets

Because of the intricate crosstalk between various immune cells, it is plausible to manipulate versatile targets by one nanoplatform. Huang et al. developed an orally delivered polymeric metformin (Polymet) that notably reinforced αPD-L1 therapy (122). The underlying mechanism of Polymet involved reprograming the immunosuppressive TME *via* adenosine 5′-monophosphate activated protein kinase (AMPK) pathway and mammalian target of rapamycin (mTOR) pathway, as well as lifting the abundance of anti-tumoral *Lactobacillus* in CRC tissues. In comparison with this single-mode therapy, it is more preferable to design multi-modular nanodrugs that counteracted several immune suppressors in TME. To this end, Jiang et al. developed a versatile nano-modulator, GSZMP. SiRNA targeting IDO-1 (siIDO) and gemcitabine (GEM) were co-encapsulated in a nanocage composed of Zinc 2-methylimidazole (ZIF-8) metal organic frameworks (MOFs) (123). The surface of drug-loaded nanocage was further tattooed with MnO_2_ mineralization and electrostatically modified with αPD-L1 or anti-CD47 antibody (αCD47) for the treatment of TNBC and colon adenocarcinoma (COAD), respectively. MnO_2_ catalytically generated O_2_ to alleviate hypoxia in TME, which promoted the repolarization of TAMs into M1 phenotype. GEM selectively depleted MDSCs, and siIDO inhibited the activity of Tregs. Overall, GSZMP reversed the “cold” TME in a multi-pronged pathway, which effectively potentiate ICB therapy (**Figure 4B**).

### 2.4 Pre-sensitizing host immune system

Tumor vaccines exhibit unique prophylactic effect against tumorigenesis and have showed combinatory therapeutic effect with immune checkpoint therapies (154–156). The aim of them is to pre-sensitize the immune system before tumor expansion and generate sufficient antigen-specific T cells, which creates a condition for subsequent invigoration of these T cells (157). Advantages of tumor vaccines can be summarized as follows: (i) tumor vaccines greatly decrease the risk of tumorigenesis; (ii) tumor vaccines induce systemic antitumor immunity that is able to attack undetectable tumor foci and metastasis (158); (iii) tumor vaccines elicit durable immune surveillance that against tumor recurrence for a long time (159); (iv) tumor vaccines can be personalized by using autologous components, which is more favorable to address the mutation of tumor antigens (158, 160). Nowadays, several tumor-exclusive neoantigens have been identified for vaccine design, but their immunostimulatory efficiency are still limited in *in vivo* studies (161). Codelivery with adjuvants will potently enhance T-cell-spurring by tumor vaccines, wherein the contrasting different drug properties between antigen and adjuvant should be concerned (159, 162). Most tumor-specific antigens are water-soluble macromolecules, such as peptides, proteins, and long chain ribonucleic acids. TLR agonists are a class of well-studied adjuvants with multiple drug forms: polyinosinic-polycytidylic acid (poly I:C, double-stranded RNA analogue, TLR3 agonist), monophosphoryl lipid A (MPLA, lipid, TLR4 agonist), Resiquimod (R848, hydrophobic small molecule, TLR7/8 agonist), and cytosine phosphate guanidine (CpG, oligodeoxynucleotides, TLR9 agonist) (163, 164). Therefore, it is highly challenging to synchronize the pharmacokinetics of antigens and adjuvants in their codelivery.

**Figure 5:**
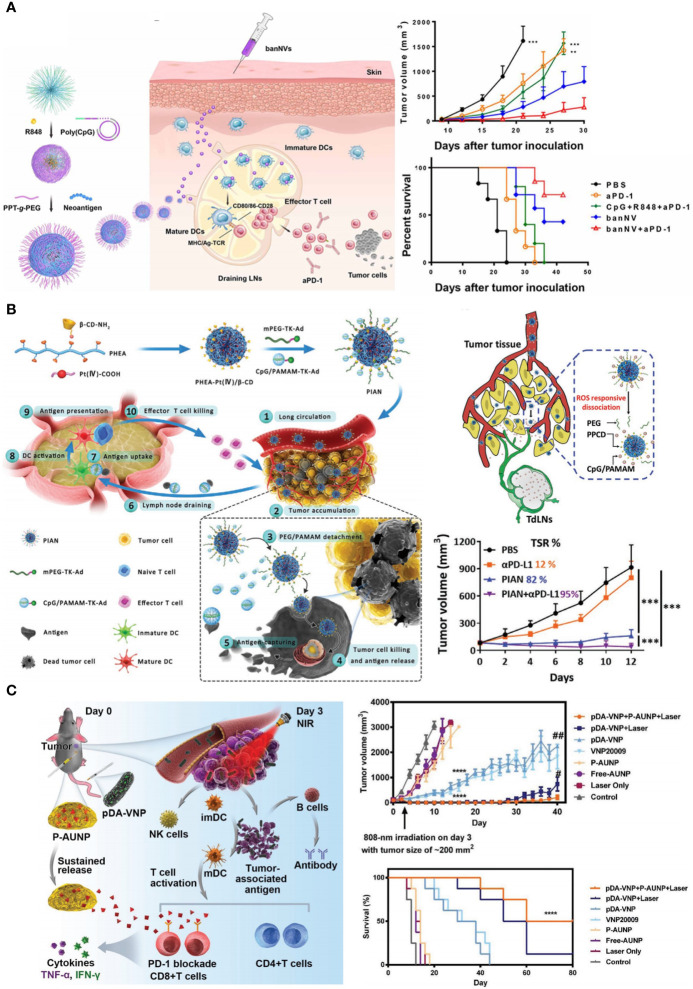
Nanotechnology pre-sensitizes host immune system to advance ICB in CRC therapy. **(A)** Left: schematic depiction of bi-adjuvant nano-vaccine (banNV) that sensitized antitumor T cells. Right: banNV remarkably facilitated αPD-1 in tumor growth inhibition and survival improvement. Reprinted with permission from reference (165). **p<0.01, ***p<0.001 versus banNV+αPD-1. **(B)** Left: schematic depiction of the programmable immune activation nanomedicine (PIAN) that generated tumor antigens *in situ* and transported these antigens to TDLNs. Right bottom: the combination of PIAN and αPD-L1 achieved considerable tumor inhibition with a tumor suppression rate (TSR) of 95%. Reprinted with permission from reference (166). ***p<0.001. **(C)** Left: schematic depiction of the combination therapy of anti-PD-1 peptide depot and bacteria-based PTT. Right: the combination therapy notably inhibited tumor growth and prolonged mice survival. Reprinted with permission from reference (167). ****p<0.0001 versus Control. ^#^ *p*<0.05, ^##^ *p*<0.01 versus pDA-VNP+P-AUNP+Laser.

Chen et al. synthesized a bi-adjuvant neoantigen nano-vaccine (banNV) that co-loaded peptide neoantigen Adpgk and two adjuvants R848 and CpG in one nanoplatform (165). Because of the activation of two TLR pathways, immunization with bi-adjuvant banNV elicited stronger T cell response than single adjuvant vaccines. As a result of immune activation, PD-1 was profoundly upregulated on Adpgk-specific CD8^+^ T cells, which led to the incomplete MC38 tumor regression after banNV treatments. Coordination withαPD-1 significantly improved the therapeutic outcomes of this bi-adjuvant vaccine therapy (**Figure 5A**). Lee et al. demonstrated the synergism of PD-L1 blockade with DC vaccine (168). They developed an immunoadjuvant nanocomplex (PSPEI-PIC) consisted of polysorbitol-co-PEI (PSPEI) polymer and poly(I:C). PSPEI-PIC assisted DC vaccines to activate tumor-specific T cells, but undesirably increased PD-L1 expression in tumor beds. Accordingly, combination of PDPEI-PIC, DC vaccine, and αPD-L1 achieved considerable therapeutic efficacy on MC38 tumor model.

Co-delivery of cytotoxic agents and immune adjuvants provides another template for tumor immunization. Chemical drugs and photosensitizers that can trigger ICD are usually used as cytotoxic agents in these nanomedicines, such as DOX, OXA, and Ce6 (169, 170). After administration, tumor cells succumbed to ICD inducers and released plenty of whole-cell antigens. These *in-situ* generated tumor antigens were immediately captured and presented by APCs with the help of adjuvant, such as imiquimod (R837, TLR7 agonist). It should be noted that cytotoxic agents worked within tumor tissues but immune adjuvants stimulated APCs in TDLNs, raising a paradox in drug delivery (41, 171). To simultaneously fulfill the site-of-actions of cytotoxic agents and adjuvants, Chen et al. developed a programmable immune activation nanomedicine (PIAN) (166). PIAN initially accumulated in tumor tissues, releasing Pt (IV) compounds (PPCD) in response to ROS for tumor killing and antigen release. Concurrently, CpG-loaded dendrimers (CpG/PAMAM) also released, capturing antigen and then transferred into TDLNs to facilitate antigen presentation. PIAN resulted in strong antitumor immune response, and completely cured 40% of colorectal tumor bearing mice in combination with PD-L1 blockade (**Figure 5B**).

Except for co-delivery with tumor antigens or antigen inducers, a sole-delivery of TLR agonists also functioned in tumor beds and exhibited synergistic effect with checkpoint inhibitors. Researches from Liu et al. revealed that intra-tumoral injection of NPs loaded with R837 or MPLA (PLGA-R837 or PLGA-MPLA) promoted DC maturation after surgery or TA of tumors, while consolidated the immunosuppressive TME (42). The anti-CTLA4 antibody (αCTLA4) were employed to inhibit Tregs. Triple therapy of TA, PLGA-R837 and αCTLA4 exerted abscopal effect to eradicate the secondary CT26 tumors and saved 100% mice from death. After primary tumor ablation, immune memory against CT26 tumors was able to lasted for 80 days. Zhang et al. prepared platelet membrane-coated nanoparticles (PNP-R848) to locally deliver R848 into tumors (172). They found the coating of platelet membrane prolonged tumor retention, improving the binding and uptake of NPs by tumor-resided immune cells. Treatment with PNP-R848 thoroughly eliminated MC38 murine colorectal adenocarcinoma and triggered a long-term immune memory that allowed mice to reject tumor rechallenge for twice.

In addition to TLR agonists, some materials derived from bacteria and virus intrinsically have adjuvant-like effects (164). Moreover, their particulate properties enable drug encapsulation. For example, Steinmetz et al. combined cowpea mosaic virus (CPMV) with αPD-1 or agonistic OX40-specific antibodies (αOX40) to combat several immunocompetent tumor models (173, 174). CPMV upregulated PD-1 on CD4^+^ and CD8^+^ effector T cells and OX40 on Tregs, which sensitized tumors to OX40 agonists and PD-1 inhibitors. In the CT26 colon cancer model, combination of αOX40 and CPMV realized better therapeutic outcomes than αPD-1+CPMV. Similar results were obtained in ovarian tumor and B16F10 melanoma models. Sun et al. are devoted to exploit *Salmonella* Typhimurium as the drug carrier for tumor immunotherapy (167, 175, 176). *Salmonella* has intrinsic tumor-homing capability, and it can release several pathogen-associated molecular patterns (PAMPs) such as flagellin and LPS to stimulate immune cells *via* TLR pathways (175). Many strategies have been developed to engineer *Salmonella* as a nanocarrier, including genetic modulation, surface modification with therapeutic agents or NPs (**Figure 5C**) (167), as well as extracting their bacterial outer membrane vesicles (OMVs) as the adjuvant to coat nano-vaccines (177, 178). These pathogen-mimicking strategies offer a simple method to achieve colocalization of antigen and adjuvant in drug delivery, and strongly correlate with the peptide-based local PD-1 blockade therapy.

## 3 Conclusions and perspectives

Although ICB-based immunotherapy has revolutionized CRC treatment, challenges such as side effects, tumor metastasis, low response rate, and therapy resistance remain in clinic. Application of nanotechnology might be ground-breaking. This review systematically discusses the current strategies that utilize nanotechnology to potentiate CRC therapy in combination with ICIs, wherein four main mechanisms are involved, including increasing delivery efficiency of ICIs, reinforcing tumor immunogenicity, reprogramming TME, and directly initiating immunity. In coming decades, we hope to witness the progress of more advanced CRC immunotherapies. Further researches are required to establish regimens that can benefit more CRC patients.

## Author contributions

ZL, YX, and YZ searched literatures and drafted the manuscript. ZL and XK designed the topic and revised the manuscript. All authors contributed to the article and approved the submitted version.

## Conflict of interest

The authors declare that the research was conducted in the absence of any commercial or financial relationships that could be construed as a potential conflict of interest.

## Publisher’s note

All claims expressed in this article are solely those of the authors and do not necessarily represent those of their affiliated organizations, or those of the publisher, the editors and the reviewers. Any product that may be evaluated in this article, or claim that may be made by its manufacturer, is not guaranteed or endorsed by the publisher.
